# High-performance, high energy density symmetric supercapacitors based on δ-MnO_2_ nanoflower electrodes incorporated with an ion-conducting polymer†

**DOI:** 10.1039/d4ra05670a

**Published:** 2024-11-08

**Authors:** Shrishti Sharma, Gurpreet Kaur, Bhargab Sharma, Buddu Nagasiva Saiteja, Anshuman Dalvi

**Affiliations:** a Department of Physics BITS Pilani-Pilani Campus RJ-333031 India adalvi@pilani.bits-pilani.ac.in; b Department of Chemistry BITS Pilani-Pilani Campus RJ-333031 India

## Abstract

The present work investigates liquid-based and liquid-free supercapacitors assembled using δ-MnO_2_-nanoflower-based electrodes. An optimized electrode composition was prepared using acetylene black (AB), a polymer (PEO), a salt (LiClO_4_), and δ-MnO_2_ and used for device fabrication. The composite electrode was tested against a liquid electrolyte and a ‘liquid-free’ composite solid polymer electrolyte (CSPE) membrane. In a three electrode geometry, with 1 M solution of LiClO_4_ as an electrolyte, the specific capacitance of the electrode was found to be ∼385 F g^−1^, with a specific energy of ∼23 W h kg^−1^ and specific power of ∼341 W kg^−1^ (at 1 mA, 1 V). Dunn's method confirmed that the charge storage process was predominantly pseudocapacitive. When the device was assembled in a two-electrode Swagelok cell, a stable specific capacitance of ∼216 F g^−1^ was observed with a specific energy of 30 W h kg^−1^ and a specific power of 417 W kg^−1^. The supercapacitors exhibited stable performance up to ∼7000 cycles with ∼90% capacitance retention and ∼97% coulombic efficiency. A combination of these cells could light two white light-emitting diodes (LEDs, 3 V) for at least ∼10 minutes. Further, all-solid-state supercapacitors (ASSCs) were fabricated using a Li^+^ ion (CSPE) membrane. The ASSCs exhibited a specific capacitance of ∼496 F g^−1^ after ∼500 cycles, with a specific energy and power of ∼19 W h kg^−1^ and ∼367 W kg^−1^, respectively. The investigation reveals that the electrodes are versatile and show compatibility with liquid and solid electrolytes. The polymer in the electrode matrix plays an important role in enhancing device performance.

## Abstract 2

The present work investigates liquid-based and liquid-free supercapacitors assembled using δ-MnO_2_-nanoflower-based electrodes.

## Graphical abstract

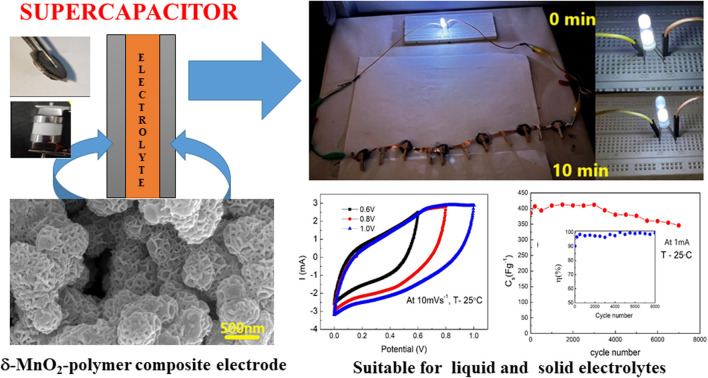

## 1 Introduction

Supercapacitors are classified into electric double-layer capacitors (EDLCs) and pseudocapacitors. While EDLCs are typically high surface area activated carbon-based devices, pseudosupercapacitors mostly use transition metal oxide-based electrodes that transfer charge *via* a faradaic process.^1^ Pseudosupercapacitors store energy *via* (i) an intercalation process leading to bulk redox reactions and (ii) surface redox reactions. These unique charge storage mechanisms lead to higher capacitance than that of EDLCs. Pseudosupercapacitors utilize both electrostatic and faradaic charge storage processes,^2^ and their cycling stability lies somewhere between that of EDLCs and batteries.

To improve the specific capacitance of pseudocapacitors, transition metal oxides (TMOs) and their hydroxides and sulfides, *e.g.* MnO_2_,^3^ RuO_2_,^4^ NiO, Ni(OH)_2_, Co(OH)_2_, Co_3_O_4_, V_2_O_5_, WO_3_, MoO_3_, and Ni_3_S_2_, as well as nitrides (VN and TiN)^5^ have been extensively used. In addition, conducting polymers, *e.g.* polyaniline (PANI)^6^ and polypyrrole (PPy),^7^ have been widely explored owing to their rapidly changing valence states during charging-discharging.^8^ Among the various TMOs, the Mn–O system possesses a wide range of structures with various stoichiometries, *e.g.* MnO, Mn_3_O_4_, Mn_2_O_3_, and MnO_2_. Moreover, within a single stoichiometry, it exists in a variety of phases (pyrolusite β, ramsdellite R, hollandite α, intergrowth γ, spinel λ, and layered δ-MnO_2_).^9^ MnO_2_ has also drawn attention because of its cost-effectiveness, low toxicity, availability, and a high theoretical pseudocapacitance value of ∼1380 F g^−1^.^10,11^

Synthesizing MnO_2_ with different nanostructures, such as nanowires,^12^ nanobelts,^13^ and nanospheres,^12^ is quite effective in improving specific capacitance. However, the actual capacitance value of MnO_2_ under supercapacitor conditions is seen to be much lower than the theoretical value (∼1380 F g^−1^), which is a significant hindrance to its practical applications.^13,14^ Among various stoichiometries, birnessite type δ-MnO_2_ is considered promising.^15^ It stands out because of its 2-D layered structure that exhibits nontoxic characteristics. Its ion exchange properties are quite effective, and it has a wide natural occurrence that makes its applications economical and cost-effective. Further, the δ-MnO_2_ in a nanoflower-like morphology provides sufficient surface exposure to the mobile ions for electrochemical reactions due to plenty of electroactive sites. Due to this, the electrochemical performance of δ-MnO_2_ nanoflower-based devices is substantially improved.^16^

An electrolyte compatible with the pseudocapacitive electrodes is another essential component that significantly influences the electrochemical performance. The incompatibility of the electrode–electrolyte interface leads to poor charge storage efficiency and instability in charge–discharge cycles. Thus, by tailoring the MnO_2_ structure *via* introducing conductive agents, *e.g.*, CNT,^17,18^ and graphene,^15^ and incorporating conducting polymers, *e.g.*, PANI,^19^ the electrode conductivity was substantially increased. The use of such additives also led to long cycling stability.^18^ Furthermore, incorporating materials with high electronic conductivity reduces the electron transfer resistance and boosts the electrochemical reactions. Hence, the tailored pseudo-capacitive electrodes open new avenues to enhance the activity of MnO_2_ active sites for the intercalation and de-intercalation of mobile salt ions.

The existing literature on MnO_2_-based supercapacitors suggests that there is still scope for improvement in the electrical properties of MnO_2_, given its low electronic conductivity and inadequate utilization of surface area. Furthermore, the ‘liquid-free’ all solid-state supercapacitors developed so far^20–24^ are predominantly of EDLC type and pseudo-capacitors using a solid electrolyte have not been explored to the best of our knowledge. For ASSCs, the electrode matrix should also exhibit adequate ionic conductivity for a smooth charge transfer across the interface.^25^

The δ-MnO_2_ nanoflower^26,27^ has been chosen in the current work as an active material in the electrode for the supercapacitors due to the above-discussed reasons. This work attempts to effectively utilize δ-MnO_2_ in nanoflower-like form in liquid electrolyte and solid polymer electrolyte-based supercapacitors. Several compositional changes in the electrode matrix have been attempted essentially to improve (i) the electronic conductivity, (ii) ionic conductivity for increasing the accessibility of mobile ions (performing capacitive action) to the MnO_2_ surface, and (iii) chemical compatibility with electrolytes. Thus, the electrode composition is tailored by introducing an ion-conducting polymer instead of a conventional binder (*e.g.*, PVDF, *etc.*) and an electronically conducting filler (acetylene black). It is thus demonstrated that addition of such an ion-conducting polymer in the δ-MnO_2_ nanoflower electrode leads to high-performance parameters not only for liquid electrolytes but also for solid polymer electrolytes. Composite solid polymer electrolyte (CSPE) membranes have recently been tested in electric double-layer supercapacitors^20–25^ and, in this study, examined for pseudo-capacitive applications.

The novel δ-MnO_2_ nanoflower-polymer electrolyte-based supercapacitors demonstrate high specific capacitance and specific energy with excellent coulombic efficiency with low equivalent series resistance (ESR) during long cycling. Using a liquid electrolyte, the supercapacitors with δ-MnO_2_-polymer electrodes were assembled and tested in 3-electrode and 2-electrode geometries. The novel electrodes were subsequently tested in ASSCs, which led to a promising performance.

## 2 Experimental

### 2.1 Preparation of electrodes with δ-MnO_2_ nanoflowers

**Fig. 1:**
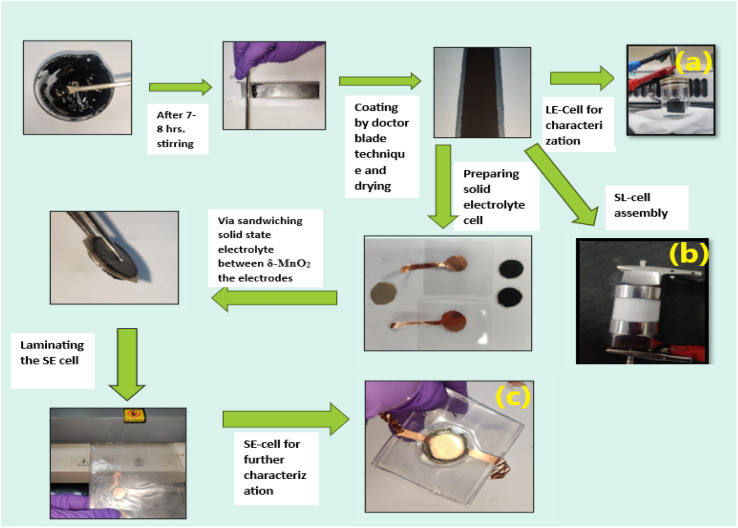
Process for making δ-MnO_2_-nanoflower-polymer electrodes and supercapacitors. Abbreviations: LE-cells: liquid electrolyte based cells in a 3-electrode setup, SL-cells: supercapacitors assembled in a Swagelok 2-electrode geometry, and SE-cells: all-solid-state supercapacitors assembled in a 2-electrode geometry.

The δ-MnO_2_ nanoflowers were synthesized by a hydrothermal route as described previously.^27^ The electrode was prepared using MnO_2_ nanoflowers as an active material and acetylene black as a conductive agent. A salt (LiClO_4_) and polymer (PEO, MW ∼ 300 000) were added to the electrode matrix. Thus, no conventional binder was added to the electrodes.^28^ In a typical compositional (wt%) ratio, *i.e.*, 65 MnO_2__5LiClO_4__10AB_20PEO, the constituents were mixed in *N*-methyl pyrrolidone (NMP) solvent. A thick slurry was obtained after ∼8 hours of stirring, which was coated on a graphite sheet (thickness ∼0.5 mm) using the doctor blade technique.^29^ The coated sheets were dried overnight at ∼100 °C in a vacuum oven, and first tested as a working electrode using a liquid electrolyte (1 M LiClO_4_) 3-electrode setup. Further, for the 3-electrode cells, the reference electrode and the counter electrode were Ag/AgCl and platinum, respectively. After ensuring satisfactory performance, a two-electrode geometry in a Swagelok cell was studied (Fig. 1). For this assembly, the electrolyte (1 M LiClO_4_) was soaked in a glass fiber (∼15 mm diameter) rod separator of thickness ∼0.2 mm. Further, for all-solid-state supercapacitors (ASSC), a composite polymer membrane of thickness ∼0.2 mm was used as the solid electrolyte and these cells were fabricated in a two-electrode geometry^24^*via* hot-roll lamination.^23,24^ The electrode size for ASSCs was ∼14 mm.^20,23,24^ The fabrication steps for various cells are described in Fig. 1.

The liquid electrolyte used was a 1 M LiClO_4_ aqueous solution. For the all-solid-state supercapacitors (ASSCs), a polymer membrane of composition 5LiClO_4__95{PEO_0.6_ LATP_0.4_} (LATP, Li_1.3_Al_0.3_Ti_1.7_(PO_4_)_3_) was used as an electrolyte. The electrolyte membrane composition is abbreviated as 40LATP (Fig. 1). These supercapacitors with an electrode diameter of ∼14 mm were thus fabricated using a hot roll lamination route as described in our previous works using the as prepared δ-MnO_2_ coated graphite sheet electrodes.

The supercapacitors in a 3-electrode setup, 2-electrode Swagelok geometry, and 2-electrode geometry with the solid polymer electrode membrane are hereafter abbreviated as LE-cell, SL-cell, and SE-cell.

Electrodes were characterized using various techniques. Field emission scanning electron microscopy (FESEM) and energy dispersive X-ray spectroscopy (EDS) were carried out using an FEI-Apreo-S to study the surface morphology. An X-ray diffractometer (Rigaku Miniflex II) with a Cu-Kα wavelength of 1.54 Å was used to determine the crystal structure. Further, X-ray photoelectron spectroscopy (XPS) (Thermo Scientific K-α) and Raman spectroscopy (Horiba Omega Scope) were performed to confirm MnO_2_ formation. The pore size and pore distribution of δ-MnO_2_ nanoflowers were evaluated by using a Belsorp-MINI X BET surface area analyzer. Electrochemical measurements were performed to investigate the performance of the supercapacitors. The cells were characterized using an electrochemical workstation Autolab M204.

## 3 Results and discussion

### 3.1 FESEM analysis

**Fig. 2:**
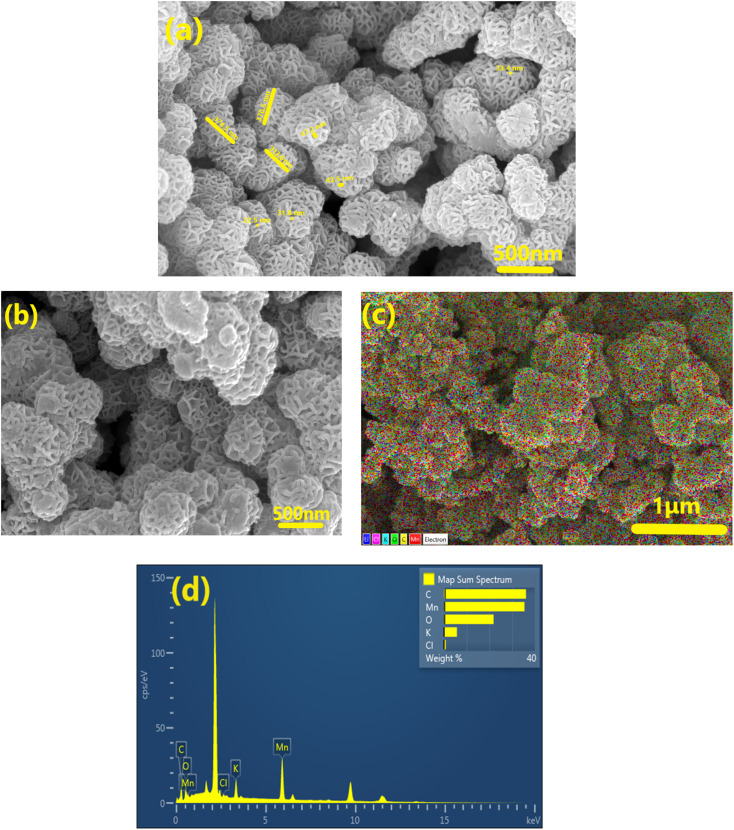
FESEM investigations of (a) pristine δ-MnO_2_ nanoflowers and (b) MNF-polymer electrodes and (c) EDX with color spectrum and (d) EDS spectrum of MNF-polymer electrodes at 1 μm.

Typical FESEM images of the as prepared δ-MnO_2_ are displayed in Fig. 2, which suggest that the δ-MnO_2_ morphology primarily exhibits flower-like patterns. The nanoflowers appear similar in morphology to earlier investigations.^27,30^ The flower-like structure is highly porous. The MnO_2_ nanoflowers (MNF) with an approximate diameter of ∼380 ± 20 nm appear to comprise numerous interconnected, thin petals with wrinkled and smooth textures. In addition, a single nanoflower petal appears to be of thickness in a range of 33 ± 5 nm, as seen in Fig. 2(a). It is also noteworthy that the nanoflowers have several macropores within the petals. Such a structure enhances reactivity and enables potential use in supercapacitors.

Fig. 2(b) shows the FESEM image of the composite electrode surface (having ∼60 wt% of δ-MnO_2_ nanoflowers). It is evident that δ-MnO_2_ nanoflowers in the presence of acetylene black, and a small amount of polymer result in a dense composite. Adding the host polymer (PEO + salt) to the electrode does not disturb the flower pattern, but the pores appear to be clogged with the polymer. Such mixing is expected to establish a smooth contact with the electrolyte. The SEM-EDX mapping (C, O, Mn, K, and Cl) image of elements on the electrode surface is shown in Fig. 2(c). Elements are distributed evenly in the matrix. Fig. 2(d) shows the EDS spectrum of the electrode. The elemental analysis confirms the presence of Mn, C, and O in large amounts. Further, the elements K and Cl also exist in the matrix, but in a small amount.

### 3.2 XRD characterization

**Fig. 3:**
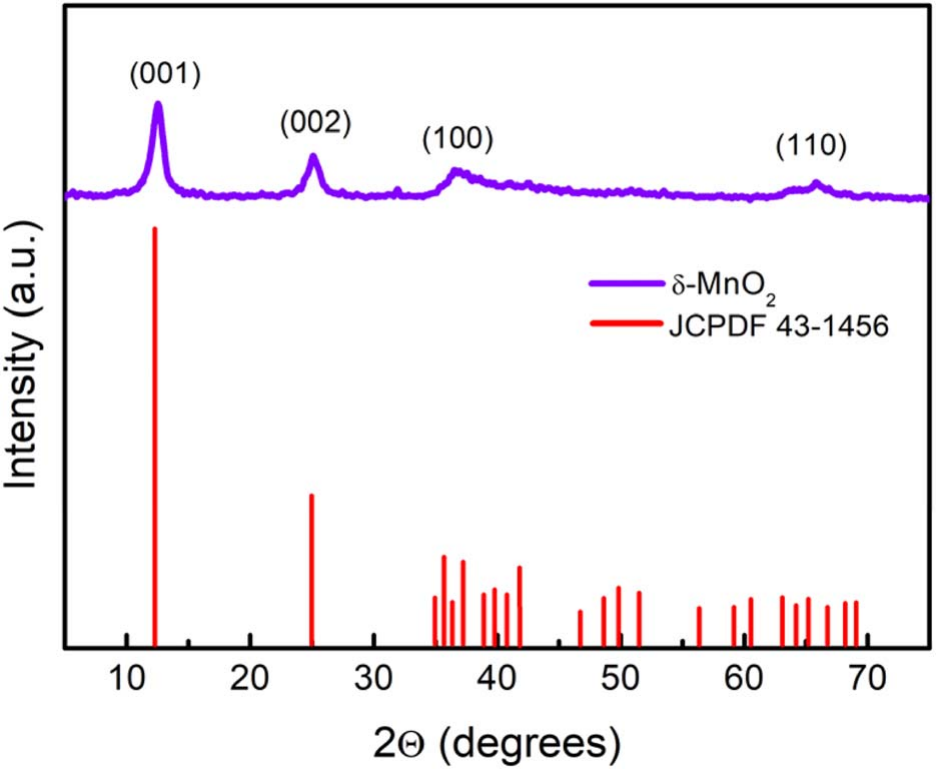
XRD pattern identified as the δ-MnO_2_ layered structure as per JCPDF card no. 43-1456. The crystallite size was calculated using the Scherrer formula 
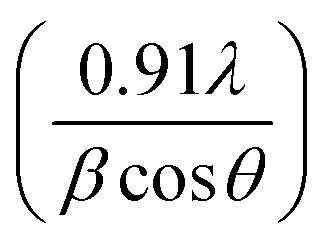
, where *λ*, *θ*, and *β* represent the X-ray wavelength, Bragg's angle and FWHM of the peak.

The wide scan patterns for the as prepared δ-MnO_2_ (Fig. 3) display the standard peaks^31^ that match well with those reported in earlier investigations^15^ and JCPDF file 00-043-1456 corresponding to birnessite-type δ-MnO_2_. Thus the patterns confirm the presence of the desired phase in high purity. The clearly defined diffraction peaks at 12.53, 25.14, 36.6, and 65.21 (2*θ*) in the MNF XRD pattern can be assigned to (001), (002), (100), and (110), respectively. The (001) and (002) planes correspond to the layered structure of δ-MnO_2_, but the wider (100) plane denotes the creation of a water–MnO_2_ interlayer and crystallisation of water.^31^ The diffraction peaks clearly indicate that the δ-MnO_2_ in this form has good crystallinity. The average crystallite size using the Debye–Scherrer relation is found to be ∼7 nm.^32^ The broadness of peaks apparently complements the small crystallite size. The basal plane spacing for the first (001) phase is ∼0.72 nm, also confirming a birnessite type layered structure as reported previously.^33^

### 3.3 X-ray photoelectron spectroscopy

**Fig. 4:**
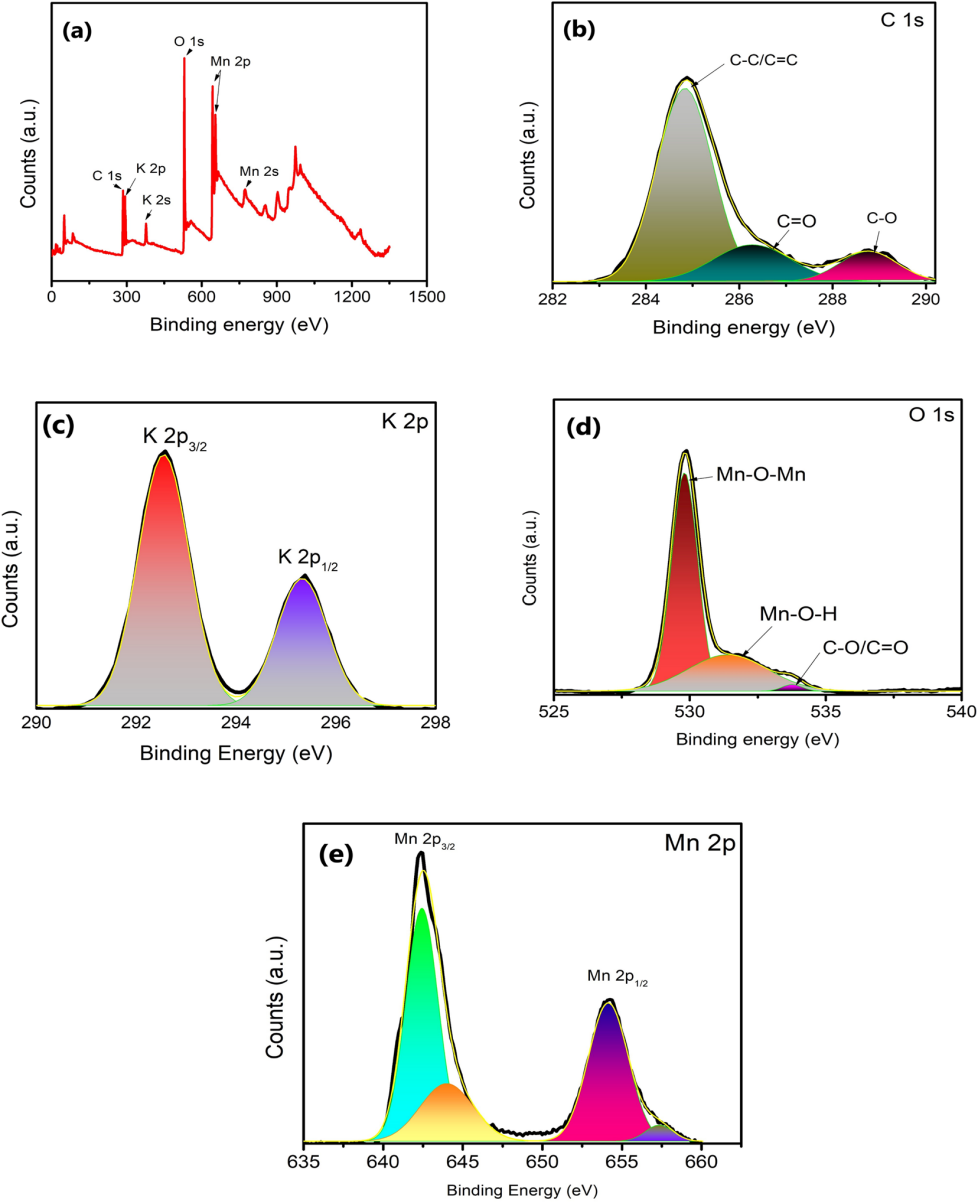
(a) XPS survey spectra for δ-MnO_2_ nanoflowers and fitting corresponding to (b) C, (c) K, (d) O and (e) Mn elements.

The surface structure of δ-MnO_2_ nanoflowers was studied with XPS as shown in Fig. 4. The presence of C, O, Mn and K in the survey scan (Fig. 4(a)) of XPS complements the EDS analysis. The XPS deconvolution was studied using the Advantage 2.0 program. From the C 1s scan in Fig. 4(b), the main peak in the C 1s area lies at 284.8 eV and is attributable to the C–C and C=C bonds.^34^ Further, C=O and C–O bonds are represented by two additional weak peaks with centers at 288.7 and 286.5 eV, respectively.^34^ Additionally, the K 2p XPS spectrum was also captured. Two prominent peaks at 292.5 and 295.3 eV, which are shown in Fig. 4(c) as K 2p_3/2_ and K 2p_1/2_, respectively, further demonstrate that potassium (in K^+^) is still present in the interlayers of birnessite-type MnO_2_. Further, the O 1s area (Fig. 4(d)) can also be split into three peaks at 529.8, 531.7, and 533.4 eV, which are readily attributed to the Mn–O–Mn, Mn–O–H, and C–O/C=C peaks, respectively. These binding energy values suggest that δ-MnO_2_ exists in the hydrated form due to the presence of water molecules on the layered surface.^31^ Two major peaks witnessed in the Mn 2p spectrum (Fig. 4(e)) are attributed to Mn 2p_3/2_ (642.32 eV) and Mn 2p_1/2_ (654.01 eV).^34^ The splitting width of 11.69 eV between Mn 2p_3/2_ and Mn 2p_1/2_ assigned to Mn^4+^ confirms the formation of MnO_2_ in the composite.^35^ This energy separation value (11.69 eV) is consistent with other studies^36^ which indicates that the oxidation state of δ-MnO_2_ is +4, and hence the layered structure of MnO_2_.

### 3.4 Raman spectroscopy

**Fig. 5:**
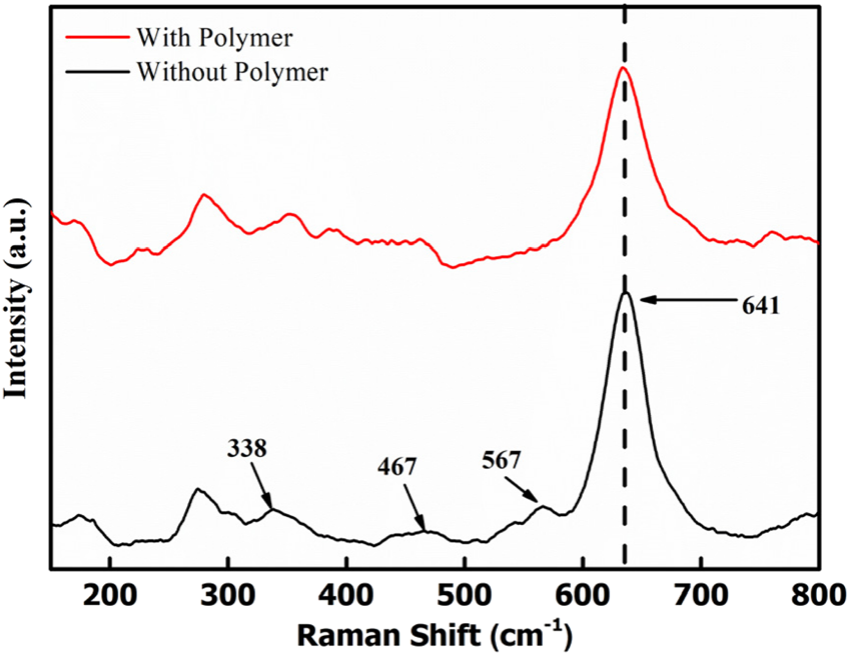
Raman spectra for δ-MnO_2_ nanoflowers with and without a polymer.

Raman spectra were obtained to examine the bonding in δ-MnO_2_ coated on the electrodes. Fig. 5 shows the Raman spectra of two samples, *i.e.*, MnO_2_ nanoflowers with PEO and without PEO. The band at 641 cm^−1^ is assigned to the A_1g_ mode, which is related to the symmetric stretching vibration (ν_2_) of the (Mn–O) bonds in the [MnO_6_] groups. Additionally, the band at 567 cm^−1^ is assigned to the symmetric stretching vibration (ν_3_) of the (Mn–O) bonds in the basal plane of the [MnO_6_] sheets, while the band at 467 cm^−1^ is attributed to the stretching vibration of the MnO_6_ group and the bending vibration in the MnO_2_ lattice.^37^ These three Raman bands for MnO_2_ are very similar to those three major vibrational features of birnessite-type MnO_2_ compounds.^38^ Polymer addition does not lead to any new absorption peak, thus eliminating the possibility of any complexation. Further, the MnO_2_ peaks are intact and almost at the same position. Thus, the environment around the MnO_6_ does not change with the polymer addition.

The above results suggest that δ-MnO_2_, polymer and acetylene black stay in a separate phase in the composite and do not form a complex.

### 3.5 BET characterization

**Fig. 6:**
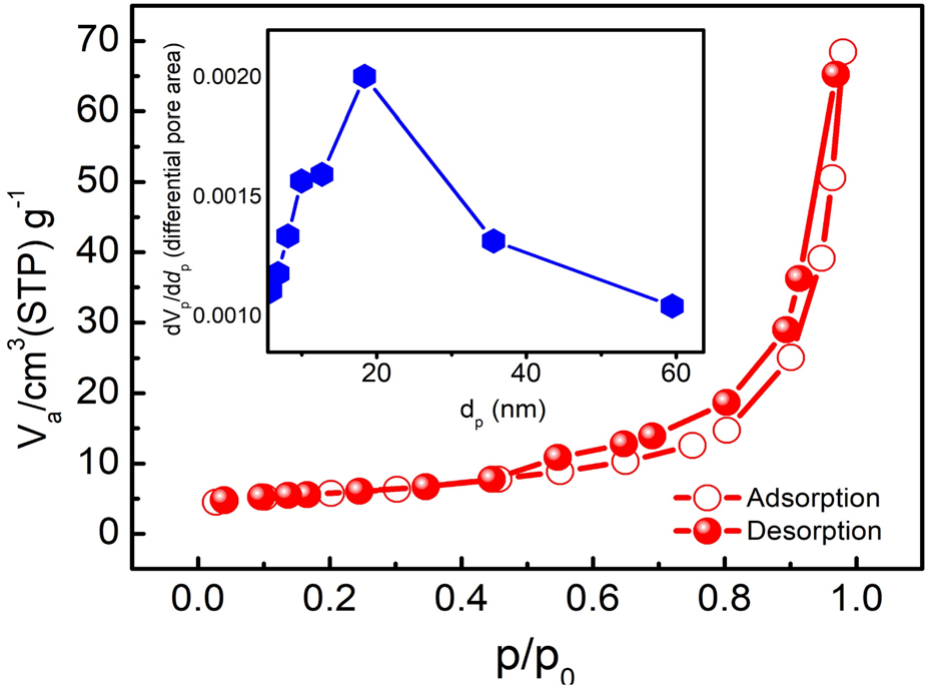
Nitrogen adsorption–desorption isotherm at 77 K and pore size distribution (inset) for the as prepared δ-MnO_2_ nanoflowers.

The nitrogen adsorption–desorption isotherm and BET plot of δ-MnO_2_ are shown in Fig. 6. For a typical sample of MNFs, closed hysteresis (above *P*/*P*_0_ ∼0.5) of type IV pattern in the desorption isotherm is observed which suggests the presence of mesopores. The hysteresis suggests slit-like pores formed due to the layered structure.^33^ As evident, the majority of the uptake takes place between the relative pressures of 0.1 and 0.95, which exhibits a uniform pore width. From the inset, the surface area and pore volume were found to be 20.8 m^2^ g^−1^ and 0.1059 cm^3^ g^−1^, respectively. The corresponding pore size distributions were determined using the Barrett–Joyner–Halenda (BJH) method and nitrogen adsorption isotherm. The result indicates that most of the pores lie in the size range of 15–25 nm. Such a large pore size offers sufficient space and therefore was found suitable for the polymer-with-salt to occupy.

The porous nanoflowers with high surface area evidently provide more active sites for the electrochemical process, hence favourable for use in charge storage applications.

### 3.6 Electrochemical characterization

For quantitative analysis various electrochemical performance parameters were evaluated *viz.* specific capacitance (*C*_s_), specific energy (*E*), specific power (*P*), coulombic efficiency (*η*) and equivalent series resistance (ESR).^39–42^ Using GCD cycles, the total resulting capacitance (in Farads) of the device is obtained from the discharge time (Δ*t*) in seconds, discharge current (*I*) (amperes), and voltage window Δ*V* (volts) of the discharge cycle (excluding IR drop) as1
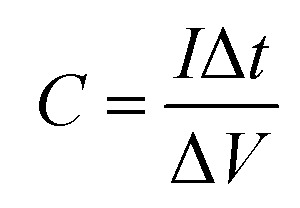


The specific capacitance per electrode (F g^−1^) is given by2
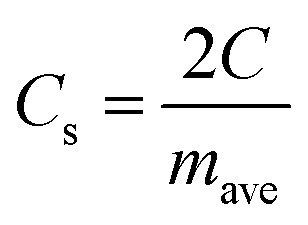
where *m*_ave_ is the average mass of the active material (in grams) on a graphite electrode.

The specific energy (W h kg^−1^) for the cell is given by3
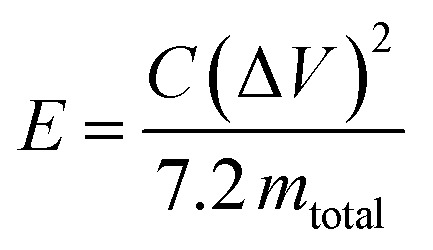
where *m*_total_ is the total mass (kg) of the active material on the electrodes.

Further, the specific power (W kg^−1^) of the device is calculated using specific energy as4
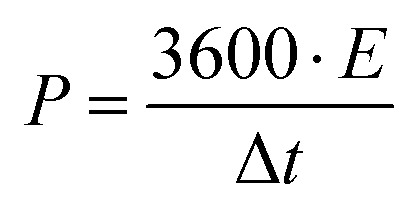


The equivalent series resistance (ESR) is obtained from the initial voltage drop Δ*V*_IR_ and discharge current *I* (A) as5
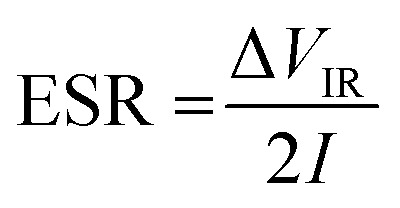


Furthermore, the coulombic efficiency, obtained from charging and discharging times, is given by6
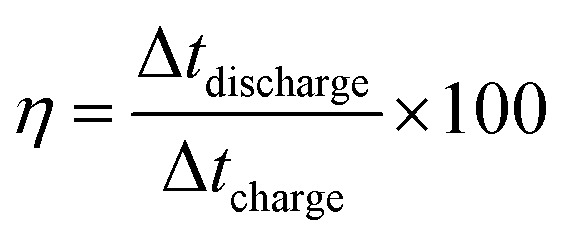


Alternatively, from the CV scans, the specific capacitance (in Farads) per electrode is calculated using the following equation:7
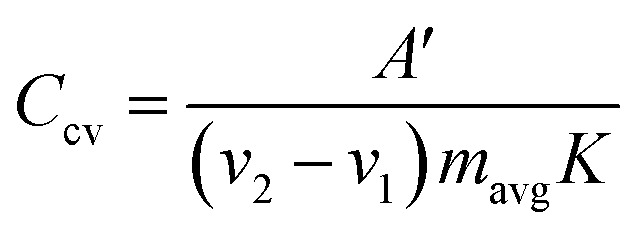
where *A*′ is the area under the CV curve, *K* is the scan rate and *v*_1_ and *v*_2_ are the voltage limits.

The resulting areal capacitance (F cm^−2^) of the cell is obtained using the following equation:8
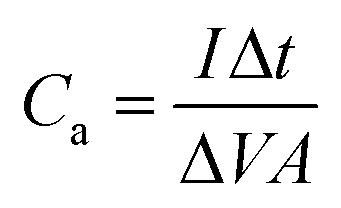
where *A* is the electrode area (cm^2^).

#### 3.6.1 Electrode characterization (LE-cell)

**Fig. 7:**
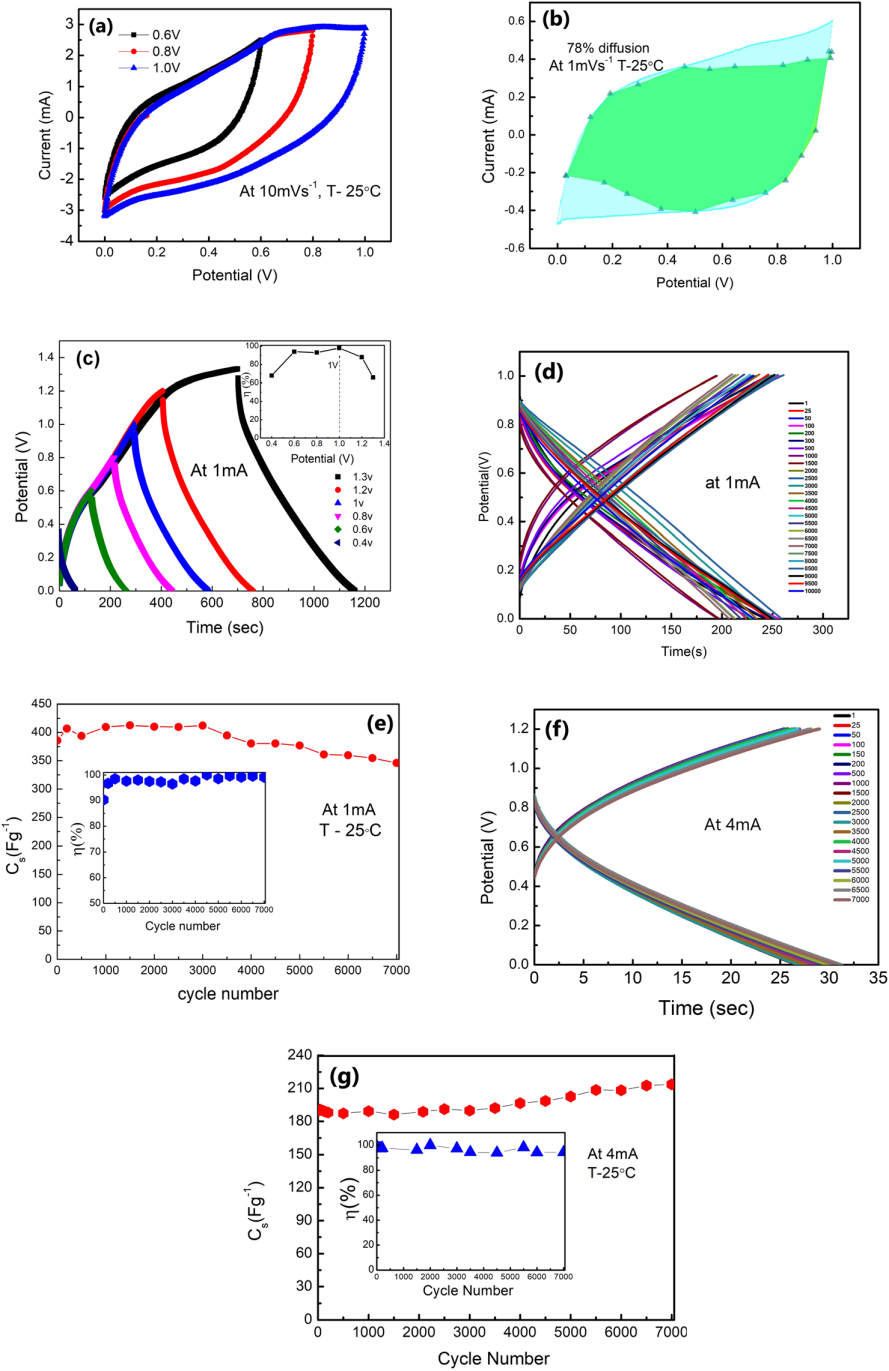
Performance of LE-cells: (a) CV scans (10 mV s^−1^) at different voltages in a 3-electrode geometry. (b) Dunn's method analysis shows that the region shaded in green signifies the dominance of diffusion (78%) in a CV (shaded in cyan) at 1 mV s^−1^, which consists of both diffusion and electrostatic contribution of charge storage. (c) GCD scans at different voltages ranging from 0.4 V to 1.3 V. Inset: *η versus* potential. (d) Potential *vs.* time at 1 mA with cycles up to 7000. (e) *C*_s_*vs.* cycle number at 1 mA. The inset shows the *η vs.* cycle number at 1 mA. (f) Potential *vs.* time at 4 mA with cycles up to 7000. (g) *C*_s_*vs.* cycle number at 4 mA. Inset: *C*_s_ as a function of cycle number at 4 mA with *η* variation.

The δ-MnO_2_-polymer electrode was first tested in a three-electrode geometry (LE-cell). Fig. 7(a) represents the cyclic voltammograms for the LE-cell at different cut-off voltages. The CV curves are shown at a scan rate of 10 mV s^−1^, and typically represent a ‘box-like’ behaviour with subtle surface redox peak-like features, attributed to voltages at which the ions intercalated and de-intercalated at the electrodes.

Using Dunn's method, the EDLC/pseudo contributions were separately evaluated.^43^ The current response at a fixed potential at the measured low scan rates (preferably 1–3 mV s^−1^) can be described as a combination of two separate mechanisms: (i) capacitive (surface) and (ii) diffusive charge storage mechanisms:

9 *i*(*V*) = *k*_1_*v* + *k*_2_*v*^1/2^

For analytical purposes, eqn (1) can be written as

10 *i*(*V*)/*v*^1/2^ = *k*_1_*v*^1/2^ + *k*_2_

Here, *i*(*V*) is the current at a given voltage, *v* is the scan rate, and *k*_1_ and *k*_2_ are the scan rate independent constants. Further, *k*_1_*v* and *k*_2_*v*^1/2^ correspond to the contributions from the surface capacitive effects and the diffusion intercalation process, respectively.^44^Fig. 7(b) illustrates the scan rate *versus* current plotted following eqn (9). A linear fit to data points determines *k*_1_ and *k*_2_ at each chosen voltage throughout the CV curve. The faradaic and non-faradaic processes that occur due to the insertion/deinsertion of Li^+^ ions at the active sites of δ-MnO_2_ nanoflowers are, therefore, differentiable upon deconvolution of the CV curves, and the surface and intercalation capacitance could be separated visibly (Fig. 7(b)). It is revealed from the shaded area in the CV pattern in Fig. 7(b) that these LE-cells are predominantly (∼78%) pseudo capacitive in nature (Fig. 7(b)).

The galvanostatic charge–discharge patterns of δ-MnO_2_ in the LE-cells were recorded at a constant current of 1 mA, with a gradual increase in the voltage. Cells have been charged up to a maximum value of 1.3 V (Fig. 7(c)). The potential with time is shown in Fig. 7(d) at 1 mA. Time decreases with the increasing cycle numbers which will complement the variation in *C*_s_ with the cycle number. The coulombic efficiency (*η*) is obtained to be ∼99% at 1 V, 1 mA. Thus, an operating voltage of 1 V is used for further studies. Further, Fig. 7(e) shows the variation of specific capacitance (*C*_s_) with the charge–discharge cycle number. The *C*_s_ values are stable, at least up to ∼4000 cycles (∼406–380 F g^−1^) with slight fading. Above ∼4000 cycles, the *C*_s_ value shows a subtle decrease, and after ∼7000 cycles, it approaches an almost constant value (∼346 F g^−1^), with nearly ∼90% capacitance retention. The inset in Fig. 7(e) shows that *η* attains a value within 96–99% during prolonged cycling.

Furthermore, GCD cycles at a relatively higher current of 4 mA (1.2 V) also show excellent stability with time (Fig. 7(f)). Even at higher currents, the shape of the GCD curve is maintained throughout cycles. The *C*_s_ value exhibits excellent stability up to ∼7000 charge–discharge cycles (Fig. 7(g)). It remains constant around 190 F g^−1^ for the initial thousand cycles. After this, it shows a slight increase (192–202 F g^−1^). Subsequently, after ∼5000 cycles, the value stabilises around 216–222 F g^−1^. The inset in Fig. 7(g) shows the coulombic efficiency with cycle numbers. Such a rise at higher discharge currents and in later GCD cycles was also reported by others.^45–47^ The initial cycles at 4 mA improve electrochemical activation, possibly leading to increased ion accessibility to the active surface area of δ-MnO_2_. The higher capacitance value for later cycles may also be attributed to an improved intercalation process and availability of complementary charges in the electrode material due to the depletion of oxygenated groups on electrodes as the reduction of δ-MnO_2_ progressed. Also, adding a host polymer in the electrode provides better accessibility of electrolyte ions to the micro and mesopores of the δ-MnO_2_ at high current density (4 mA).^45^

GCD scans for long cycles indicate that the stability of the polymer doped δ-MnO_2_ nanoflowers at 1 mA and 4 mA is appreciable at least up to ∼7000 charge–discharge cycles.

#### 3.6.2 Supercapacitor assembled in a Swagelok cell

**Fig. 8:**
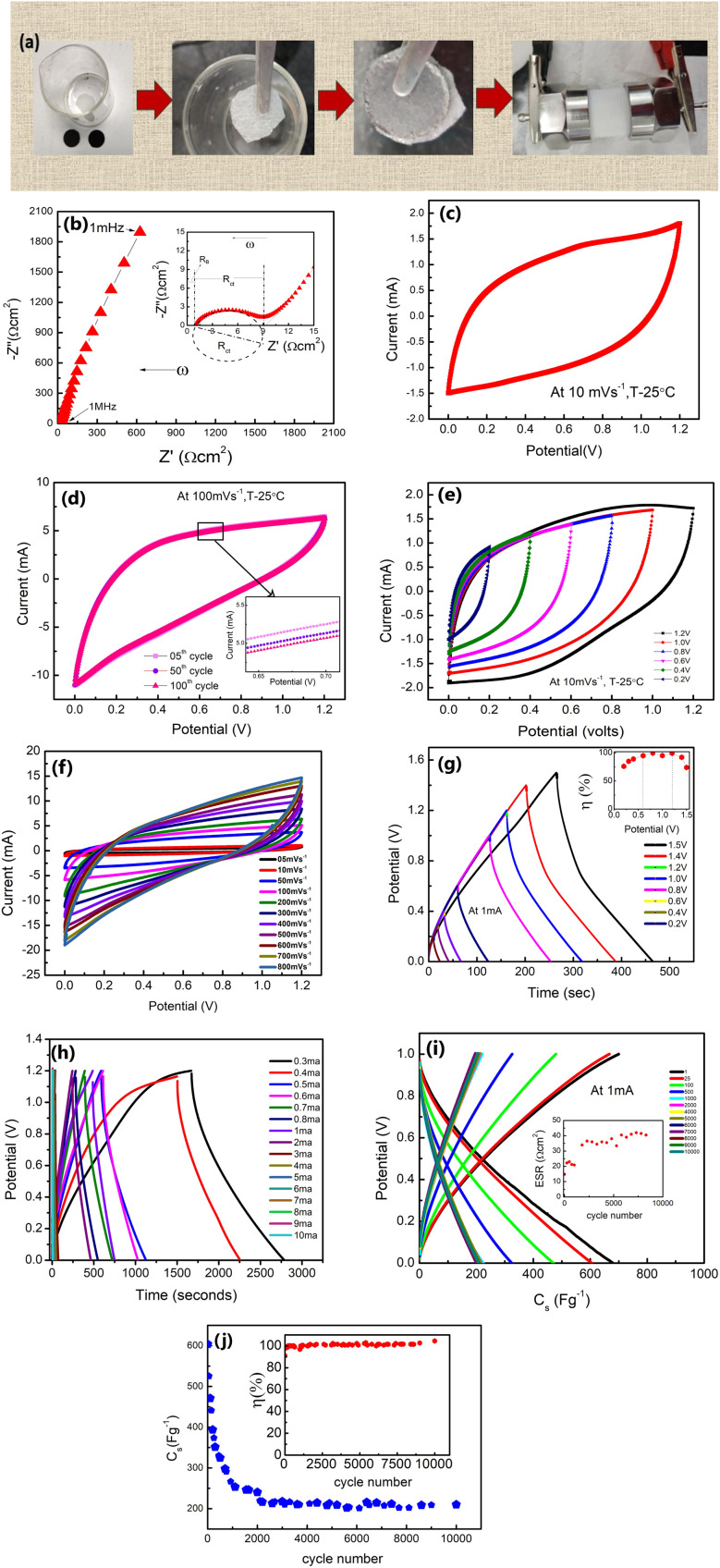
(a) Preparation route to the SL-cell with 1 M LiClO_4_ solution as the electrolyte. (b) Nyquist plot (1 mHz to 1 MHz) of the SL-cell. (c) Cyclic voltammetry scans at 10 mV s^−1^ up to 1.2 Volts. (d) CV cycles (first 100 cycles) at 100 mV s^−1^. (e) CV scans from 0.2 V to 1.2 V. (f) CV scans at different sweep rates ranging from 5 mV s^−1^ to 800 mV s^−1^. (g) GCD scans from 0.2 V to 1.5 V at 1 mA are shown; the inset shows the *η vs.* potential. (h) GCD scans at different currents (0.3–10 mA). (i) GCD scans for ∼10 000 cycles at 1 V, 1 mA. The inset shows ESR variation with the cycle number. (j) *C*_s_*vs.* the cycle number. Inset: variation in *η* with the cycle number.

The assembly process for the Swagelok cell (SL-cell) is described in Fig. 8(a). The cell exhibits a bulk resistance (*R*_b_) of 0.86 Ω cm^2^ and a significantly small charge-transfer resistance (*R*_ct_) of ∼8.3 Ω cm^2^ (Fig. 8(b)) at room temperature. Using eqn (7), the capacitance (for the electrode) at 10 mV s^−1^ is obtained to be ∼316 F g^−1^ (Fig. 8(c)). The CV scans for the first ∼100 cycles are almost overlapping (Fig. 8(d) and inset) with a typical capacitance value of ∼162 F g^−1^. A reduced specific capacitance with increasing scan rate is a typical behaviour of supercapacitors attributed to the limited penetration of electrolyte ions at higher rates.^48,49^ High scan rates also lead to the rapid movement of mobile ions, leading to an increase in the resistance that also causes the capacitance to fall. The cycles are stable up to a cutoff potential of ∼1.2 V, as shown in Fig. 8(e). The CV curves with scan rates ranging from 5 mV s^−1^ to 800 mV s^−1^ are displayed in Fig. 8(f) up to 1.2 volts, which show current variation from 0.5 mA to 15 mA. It is evident that redox reactions are not observable at high scan rates, as the electrolyte ions and the active species do not get sufficient time to interact.^48^ Due to the rapid movement of ions towards the electrode surface at a high scan rate, the shape of CV shifts to a leaf-like shape.^50^

Also, during GCD scans from 0.2 V to 1.2 V, and at 1 mA, the *η* value varies in a range of ∼73–99% (inset of Fig. 8(g)). Further, the GCD cycles are shown with current variation (Fig. 8(h)). At a lower current of 0.3 mA (0.441 mA g^−1^), the *C*_s_ reaches an exceptionally high value of ∼895 F g^−1^ with a *P* of ∼166 W kg^−1^ and *E* of ∼30 W h kg^−1^, whereas at ∼10 mA, *C*_s_ is found to be ∼196 F g^−1^ with a *P* value of ∼2413 W kg^−1^ and *E* of ∼5 W h kg^−1^. The graph in Fig. 8(i) shows GCD curves for ∼10 000 cycles. In the initial cycles, the ESR (inset, Fig. 8(i)) is approximately ∼15 Ω cm^2^ and increases to a value of 22 Ω cm^2^ after ∼1000 cycles. Subsequently, from ∼1700 to ∼10 000 cycles, the ESR still remains substantially low at ∼33–41 Ω cm^2^.

The initial rapid decline in capacitance (*C*_s_) could be linked to several factors. One possible explanation is the interaction between mobile ions and active sites on the electrode surface [50], leading to charge consumption and an early drop in capacitance. Additionally, this fading could be attributed to the partial blockage of the porous or layered electrode structure during the early stages of electrolyte ion intercalation and de-intercalation. This reduces pore accessibility to the electrolyte ions after the first few cycles. Further, in the SL-cell, the liquid electrolyte is trapped in a glassy carbon membrane which is under plunger-pressure. Initial comparison may remove excess solvent resulting in salt precipitation.^48^ A drop in the capacitance may also be due to the polarization/accumulation of ions at various interfaces, *e.g.*, grain–grain (of the electrolyte) and electrode–electrolyte boundaries.^23^

While the *C*_s_ value thus drops from ∼673 F g^−1^ to ∼350 F g^−1^ in the initial ∼200 cycles, during 250–2000 cycles, its decrease is rather gradual from ∼350 F g^−1^ to ∼250 F g^−1^ (Fig. 8(j)). Subsequently, *C*_s_ values are stable from ∼2000 to 10 000 cycles around ∼210–220 F g^−1^. The stability after ∼2000 cycles indicates the facile ionic motion in the porous interiors of δ-MnO_2_ electrodes for a large number of cycles.^14^ As shown in the inset of Fig. 8(j), the *η* maintains a high value of ∼99% consistently during these cycles.

#### 3.6.3 SE-cells: solid electrolyte-based cells with novel electrodes

**Fig. 9:**
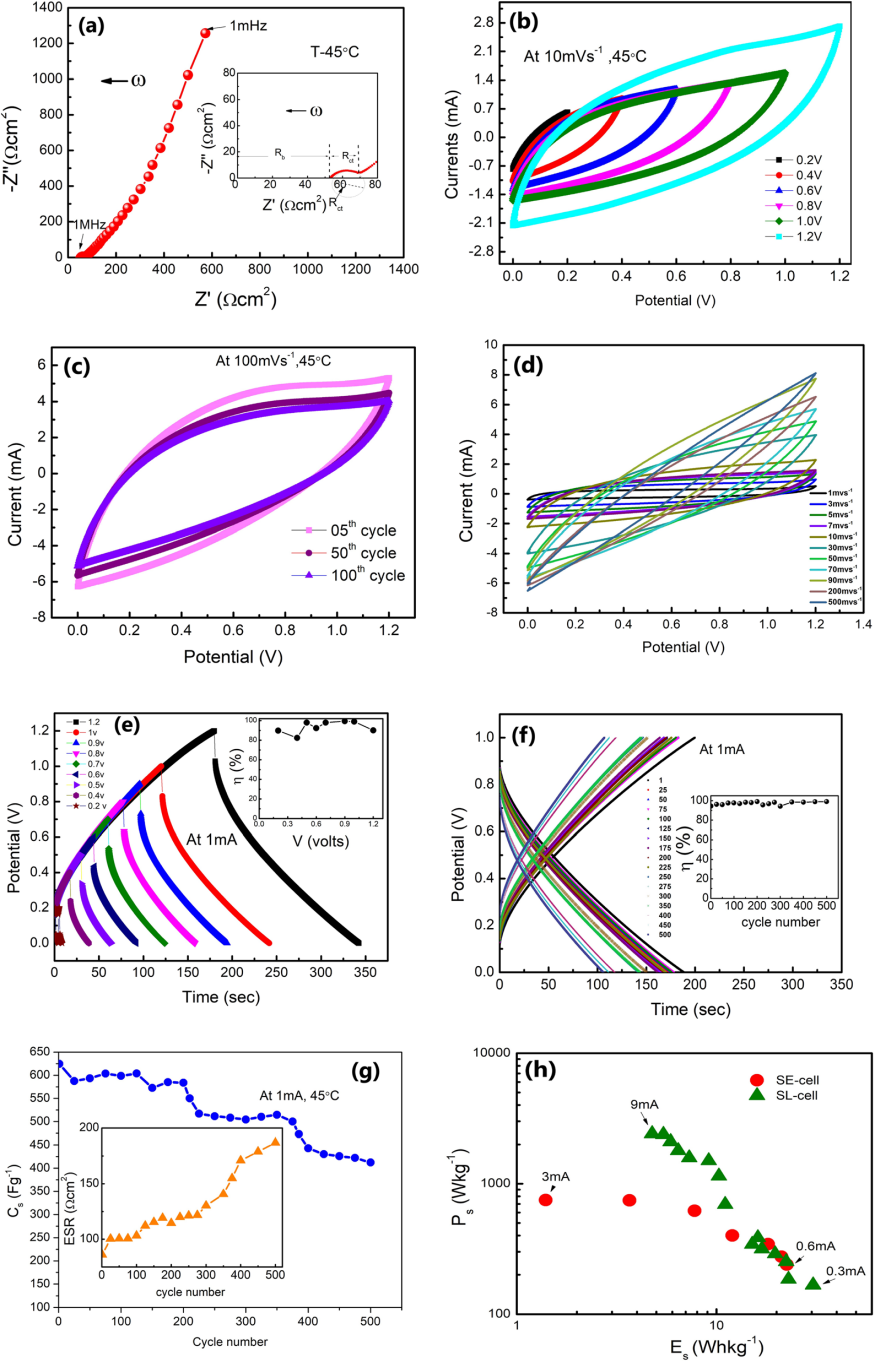
Performance of SE-cells: (a) Nyquist plots (1 mHz to 1 MHz) with the inset showing an extended scale. (b) CV scans at different operational voltages at a scan rate of 10 mV s^−1^. (c) 100 CV scans up to 1.2 V at 100 mV s^−1^. (d) CV scans at different scan rates ranging from 1 mV s^−1^ to 500 mV s^−1^. (e) GCD scans at different potential ranging from 0.2 V to 1.2 V. The inset shows *η* variation with operating voltage. (f) GCD scans for the first 500 cycles. The inset shows *η versus* the cycle number. (g) *C*_s_ with the cycle number. The inset shows the ESR variation with cycles. (h) Ragone plot for the SL cell and SE cell.

Nyquist plots (1 mHz to 1 MHz) for the SE cell with the CSPE membrane as an electrolyte is shown in Fig. 9(a). The SE-cell was also operated in a two electrode sandwiched geometry, but at 45 °C. The charge-transfer resistance (*R*_ct_) of ∼16.83 Ω cm^2^ and bulk resistance (*R*_b_) of ∼52.74 Ω cm^2^, in the impedance spectra, are remarkably low in comparison to other solid state supercapacitors reported earlier.^20–24,51^ In fact, these values are slightly higher than the corresponding values for the SL-cell. Such low ESR suggests that the electrode–electrolyte interface utilizes the high surface area of the activated carbon, and can facilitate faster charge transfer across the interface.^35^ The CV curves shown at 10 mV s^−1^ up to 1.2 Volts do not show any peak-like feature (Fig. 9(b)). Also, as illustrated in Fig. 9(c) CV scans (100 mV s^−1^) are stable up to ∼100 cycles with a slightly decreased area under the curve. Due to the solid–solid interface, the pseudo (faradaic) charge storage may also be accompanied by (i) local charge accumulation within the solid polymer electrolyte matrix and (ii) interfacial polarization at the electrode–electrolyte interface. For such supercapacitors, the CV scans at different sweep rates (1–500 mV s^−1^) exhibit currents in a range of ∼1 mA to 9 mA (Fig. 9(d)).

Further, Fig. 9(e) shows the charge–discharge cycles for the SE-cell at different voltages from 0.2 V to 1.2 V. The inset shows the variation of the *η*-value with potential, which remains close to 100% for 1 V. GCD cycles *versus* discharge time are shown in Fig. 9(f). The *η*-value is almost constant for these 500 cycles. Further, the *C*_s_ (obtained for Fig. 9(f)) is stable initially for the first 200 cycles (∼624–584 F g^−1^) and later stabilizes to ∼406 F g^−1^ after 500 cycles (Fig. 9(g)). The inset of Fig. 9(g) shows the variation of ESR values with the cycle number for the SE-cell. ESR values show a gradual increase from 86 Ω cm^2^ to ∼187 Ω cm^2^. After 500 cycles the measurements were not performed due to the rapid fall in the values. This fall could be due to natural detachment of the solid–solid interface due to different thermal expansions of the electrode and electrolyte. Possibly, such cells may show long cycling stability under adequate external pressure. More efforts are required in this direction.

Interestingly, as apparent in the Ragone plots (Fig. 9(h)), the values of specific energy (*E*) and power (*P*) coincide for lower currents with those of SL-cells, which is quite appreciable in view of the SE cell being a liquid free device.

The present results obtained using δ-MnO_2_-polymer composite electrodes are summarized in Table 1. The performance parameters for SE-cells and SL-cells are comparable. This is permissible as the SE-cells were operated at slightly higher temperatures.

**Table 1:** Performance parameters for cells with polymer-added δ-MnO_2_ electrodes, *viz.* specific capacitance (*C*_s_), areal capacitance (*C*_a_), specific energy (*E*), specific power (*P*), equivalent series resistance (ESR) and coulombic efficiency (*η*) values for SL cells^a^

Electrolyte	Cell geometry	System	*C*_s_ (F g^−1^)	*C*_a_ (mF cm^−2^)	*E*_s_ (W h kg^−1^)	*P*_s_ (W kg^−1^)	ESR (Ω cm^2^)	*η*
Liquid (1 M LiClO_4_ electrolyte)	Swagelok	Swagelok geometry (two electrode system)	665–680	140–160	27–38	400–450	40–50	94–99
			676*	149*	30*	418*	43*	97%*
Solid (40 LATP electrolyte)	Laminated	40LATP_10LiClO_4_	610–640	125–145	17–22	350–400	80–90	92–95
			624*	138*	19*	367*	86*	94%*

a The values with * correspond to the performance of a supercapacitor close to the average value.

For comparison, supercapacitors with polymer-free δ-MnO_2_ electrodes were also assembled and are reported in Table 2. A detailed analysis is given in the ESI (Fig. S1–S3).† Apparently, polymer (+salt) addition leads to superior performance of SE-cells and SL-cells. These results suggest that the presence of the polymer facilitates the Li^+^ ions to intercalate into the electrode matrix. Such a long-range diffusive transport is likely *via* the coupling of mobile Li^+^ ions with the ether oxygen of PEO. Also, for SE-cells, the presence of the polymer establishes smooth electrode–electrolyte contact and allows effective utilization of petal surface area.

**Table 2:** The performance parameters for cells without polymer δ-MnO_2_ electrodes^a^

Electrolyte	Cell geometry	*C*_s_ (F g^−1^)	*C*_a_ (mF cm^−2^)	*E*_s_ (W h kg^−1^)	*P*_s_ (W kg^−1^)	ESR (Ω cm^2^)	*η*
Liquid (1 M LiClO_4_ electrolyte)	Swagelok	460–508	102–112	17–20	400–450	55–65	85
		484*	107*	19*	412*	60*	
Solid (40 LATP)	Laminated	243–269	53–59	7–10	342–377	139–154	75
		256*	56.6*	8.77*	359*	147*	

a The values with (*) correspond to a typical supercapacitor whose performance is close to the average value.

**Fig. 10:**
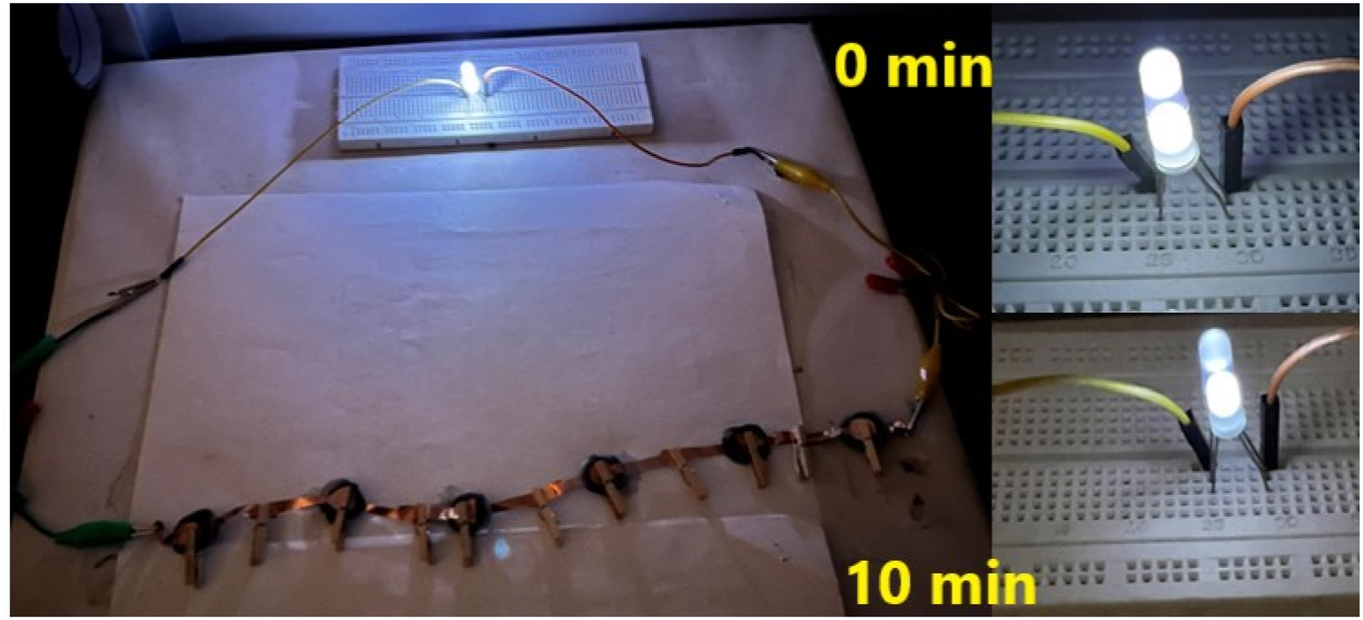
LEDs each of 3 V (∼60 mW) during the direct discharge of SL cells for 10 minutes.

The δ-MnO_2_-polymer composite electrode demonstrated applicability in both liquid and solid electrolytes. The supercapacitors in series (Fig. 10) could light two ∼3 V white LEDs with slight fading in intensity for at least 10 minutes.

The values reported in the present study are comparable to those in earlier reports. Performance is superior in terms of cycling and efficiency. The all-solid-state δ-MnO_2_-supercapacitor performance is reported for the first time (Table 3).

**Table 3:** Comparison of various performance parameters of earlier reported δ-MnO_2_-based supercapacitors with the present investigation. The operational parameters, *e.g.* temperature, discharge currents as well as electrolyte and its concentration, may vary in different studies

Electrode	Capacitance/current	Electrolyte	Important findings
MnO_2_@MnO_2_, core/shell nanostructures^52^	190.5 F g^−1^ (5 A g^−1^)	0.5 M Na_2_SO_4_	92.2% capacity retention after 20 000 cycles
MnO_2_-coated GrP (GrP/10-MnO_2_) (graphene paper), Sadak *et al.*^53^	385.2 F g^−1^, 1 mV s^−1^	0.1 M Na_2_SO_4_	82.2% capacity retention after 5000 cycles
	768.52 m F cm^−2^ (0.05 mA cm^−2^)		
MnO_2_ nanoflower, Gomaa *et al.*^54^	309 F g^−1^ (0.1 A g^−1^)	1 M Na_2_SO_4_	Capacity retention 93% over 1650 GCD cycles
Birnessite type MnO_2_ nanoflower, Zhao *et al.*^55^	197.3 F g^−1^ (1 A g^−1^)	1 M Na_2_SO_4_	Capacity retention of ∼94.6% after 1000 cycles
3-D hierarchical birnessite-type MnO_2_ nanoflowers, Yan *et al.*^56^	251.3 F g^−1^ (0.5 A g^−1^)	0.5 M Li_2_SO_4_	Capacity retention of 92.5% after 10 000 cycles
MnO_2_ nanoflowers asymmetric cell, Sun *et al.*^57^	52.9 mF cm^−2^ (0.5 mA cm^−2^)	0.5 M Na_2_SO_4_	
Sandwich structured MnO_2_/graphene^15^	240 F g^−1^ (1 A g^−1^)	0.5 M Li_2_SO_4_	Capacity retention of 96% after 1000 cycles
Carbon dots/manganese dioxide (CQDs/MnO_2_) nanoflowers^58^	210 F g^−1^ (20 A g^−1^)	1 M Na_2_SO_4_	90.3% retention rate after 10 000 cycles
δ-MnO_2_ nanoflowers doped with PEO (present work)	(i) LE-cell: 385 F g^−1^ (1.47 A g^−1^)	1 M LiClO_4_	90% capacity retention after 7000 cycles
	(ii) SL-cell: 216 F g^−1^ (1.47 A g^−1^)	Glass fiber rods soaked 1 M LiClO_4_	Coulombic efficiency 97% after 10 000 cycles
	(iii) SE cell: 496 F g^−1^ at (1.47 A g^−1^)	40LATP_10 LiClO_4_ (solid electrolyte)	Coulombic efficiency ∼94% after 500 cycles

According to the investigations mentioned above, a tentative charge storage mechanism is proposed. In supercapacitors the charge storage primarily relies on the adsorption of mobile ions supplied by the electrolyte, which is influenced by the chemical affinities of mobile ions for the electrode surface and the nature/strength of the electric double layer. In the pseudo-capacitive process, both the creation of the Helmholtz double layer and the rapid surface faradaic process contribute to the capacitance.

(i) Electric double-layer charge storage mechanism is likely on MnO_2_ surfaces. Thus, the flower petals contribute to the physisorption of some of the mobile ions. The anions (ClO_4_^−^) of the electrolyte may also contribute as a capacitor ion *via* this process.

(ii) The pseudo capacitive charge storage process may be associated with the chemisorption of mobile Li^+^ ions on the δ-MnO_2_ nanoflower petals. A probable surface induced partial charge transfer may happen as per the reaction[MnO_2_]_petal_ + Li^+^ + e^−^ → [MnOOLi]_petal/surface_

(iii)The process of Li^+^ ion diffusion between the layers of MnO_2_ may also lead to faradaic reactions involving a change of state from Mn^3+^ to Mn^4+^. It is proposed that the ion-conducting polymer present in the electrode provides pathways for the Li^+^ ions to diffuse to the interior. Such a process may lead to the following reaction:^59^MnO_2_ + Li^+^ + e^−^ → MnOOLi

Our Dunn's method analysis indicates that the process is predominantly pseudo-capacitive. Moreover, the high coulombic efficiency in the current study suggests that surface chemisorption is predominant. The tunnel size of δ-MnO_2_ is quite large (Fig. 6), which may facilitate diffusion into the interior.

The above-suggested mechanism (i)–(iii) is well-suited for liquid electrolyte-based (SL) cells. For SE-cells, local polarization may limit the diffusive transport within the layers of MnO_2_. Thus the capacitance may have contribution from surface states *via* physisorption or chemisorption. Long range diffusive motion in solid state supercapacitors is unlikely as also suggested by a high *η* value. More theoretical and experimental investigations are required to support the proposed mechanism.

## 4 Conclusions

The work may be summarised as follows:

(i) The results demonstrate that specific capacitance has significant (∼78%) contribution from pseudo-capacitive processes. However, for low operating potential (*V*_op_ < 1 V) the process appears to be electrostatic (EDLC type). For *V*_op_> 1 V, pseudo (redox) effects are visible. It is revealed that an electrochemical stability window up to 1 V is most suitable for long cycle operations, as in this limit, *η* is high, and ESR is appreciably low. CV, EIS and GCD complement each other.

(ii) Interestingly, these electrodes are quite efficient for charge storage even when a solid electrolyte and a solid–solid interface exist. In these liquid-free activated carbon-based solid-state cells, the interfacial contacts, however, degrade with time, which thus has scope for improvement. Efforts are required to engineer the solid–solid electrode–electrolyte interface for long cycling stability.

(iii) Interestingly, these electrodes are free from poorly conductive conventional binders (PVDF, *etc.*). Instead, to modify the electrode–electrolyte interface an ion conducting polymer (PEO with LiClO_4_ salt) is used in the electrode, which decreases the ESR, improves the effective surface area and provides pathways for mobile ions. This results in improved performance.

(iv) Ragone plots show promising values of energy and power comparable to and even higher than those of many previous reports. SE-cells seem suitable for low power device applications.

(v) Galvanostatic charge–discharge cycles are stable, and the coulombic efficiency is maintained at ∼98–99% with ∼70–80% capacitance retention after long cycling.

In conclusion, we have reported, for the first time, activated carbon and binder-free novel electrodes based on δ-MnO_2_ nanoflowers and an ion-conducting polymer additive. We have revealed that adding a polymer (instead of a binder) to the electrodes enhances the performance of the supercapacitor. The layered structure of δ-MnO_2_ nanoflowers, having PEO and LiClO_4_, provides pathways on the surface and inside for intercalation and deintercalation during charging and discharging. Supercapacitors using these electrodes can be prepared in hot-roll-lamination and Swagelok geometries, and can be used as liquid-based as well as all-solid-state supercapacitors. There is scope to improve the long-cycling performance of all-solid-state supercapacitors. This can be done by improving the interface. Efforts are on in this direction.

## Data availability

All data included in this work are available upon request by contact with the corresponding author.

## Author contributions

Shrishti Sharma: conceptualization, methodology, validation, formal analysis, investigation, writing – original draft. Gurpreet Kaur: formal analysis, reviewing original draft. Bhargab Sharma: formal analysis, Raman analysis, reviewing – original draft. Buddu Nagasiva Sai Teja: investigation, methodology, validation, formal analysis, Anshuman Dalvi: conceptualization, validation, writing – review & editing, funding acquisition.

## Conflicts of interest

The authors declare that they have no known competing financial interests or personal relationships that could have appeared to influence the work reported in this paper.

## Supplementary Material

RA-014-D4RA05670A-s001
