# Supplementary material for: High-performance, high energy density symmetric supercapacitors based on δ-MnO2 nanoflower electrodes incorporated with an ion-conducting polymer
Source: RSC Adv. 2024 Nov 8;14(48):35657–70. doi: 10.1039/d4ra05670a (PMC11544595; doi:10.1039/d4ra05670a)
Supplement: RA-014-D4RA05670A-s001 [file RA-014-D4RA05670A-s001.pdf]

Supplementary Information (SI) for RSC Advances.
This journal is © The Royal Society of Chemistry 2024

High-performance, high energy density symmetric supercapacitors based on δ-MnO_2 nanoflower electrodes added with ion-conducting polymer

Shrishti Sharma[a], Gurpreet Kaur[b], Bhargab Sharma[a], Buddu Nagasiva Saiteja[a], Anshuman Dalvi[a*]

[a] Department of Physics, BITS Pilani-Pilani Campus (RJ-333031) India

[b] Department of Chemistry, BITS Pilani-Pilani Campus (RJ-333031), India

*Corresponding author email: adalvi@pilani.bits-pilani.ac.in

SUPPLEMENTARY INFORMATION

We present some important findings on supercapacitors assembled with polymer-free δ-MnO_2 electrodes for a comparison. The performance parameters evaluated from the plots are given in Table 3 of the manuscript. Mass loading, other geometrical parameters are kept same for a comparison.

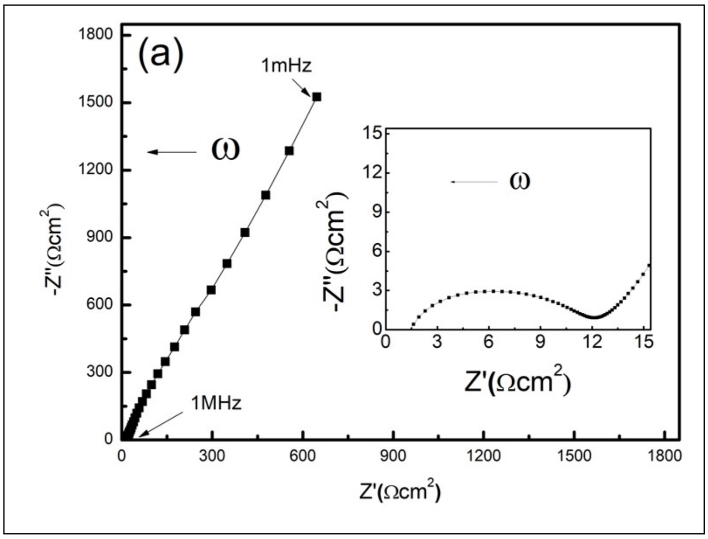

Fig. S-1(a) shows the EIS from 1mHz to 1MHz for Swagelok supercapacitor (SL-cell) in 1M $LiClO_4$. In comparison to the cells with polymer-added electrodes ESR is high (Fig. 8(a)), the low frequency tail is steeper.

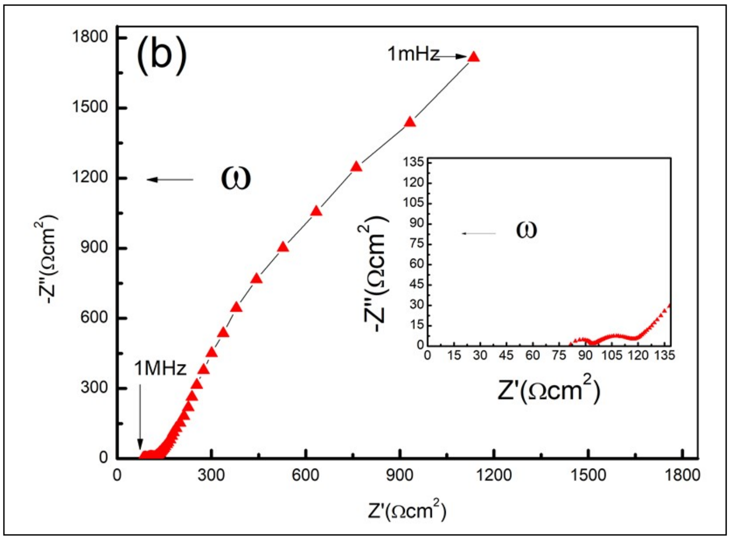

Fig. S-1(b): Performance of all-solid-state supercapacitor (SE-cells) with polymer-free electrodes: Nyquist plots (1mHz-1MHz) exhibit higher ESR value than reported for SE-cell with polymer electrode (Fig. 9 (a)). The charge transfer process is more complex in this case, as two semicircles are visible.

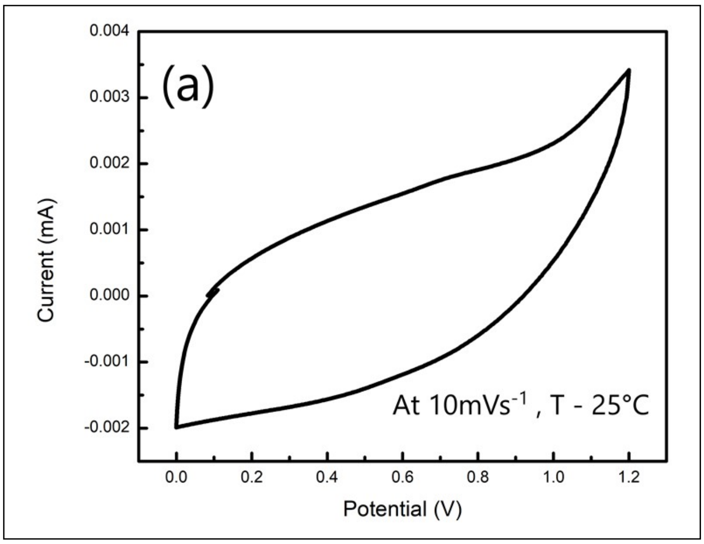

Fig. S-2 (a) CV scans at $10mVs^{-1}$ for SL-cell without polymer incorporated electrodes. The area under the curve and the corresponding capacitance is smaller than that of the polymer-added electrode based cells. Further, corresponding highest current value for the SL-cell with polymer-added electrodes is notably higher (Fig. 8 (c)).

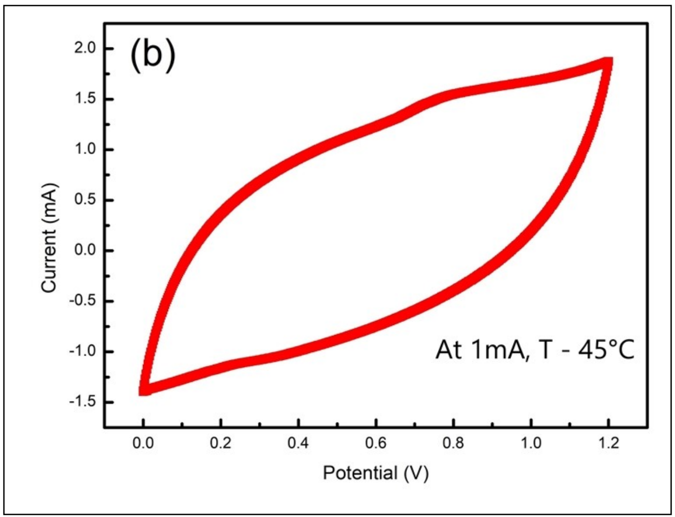

Fig. S-2(b): CV scans for all-solid-state supercapacitor (SE-cells) with polymer-free at $10mVs^{-1}$ for SE-cell. The area under the curve and the corresponding capacitance is smaller than that of the polymer-added electrode based cells. The area under the curve and the corresponding capacitance is smaller than that of the polymer-added electrode based cells. Further, corresponding highest current value for the SL-cell with polymer-added electrodes is notably higher (Fig. 8 (c)).

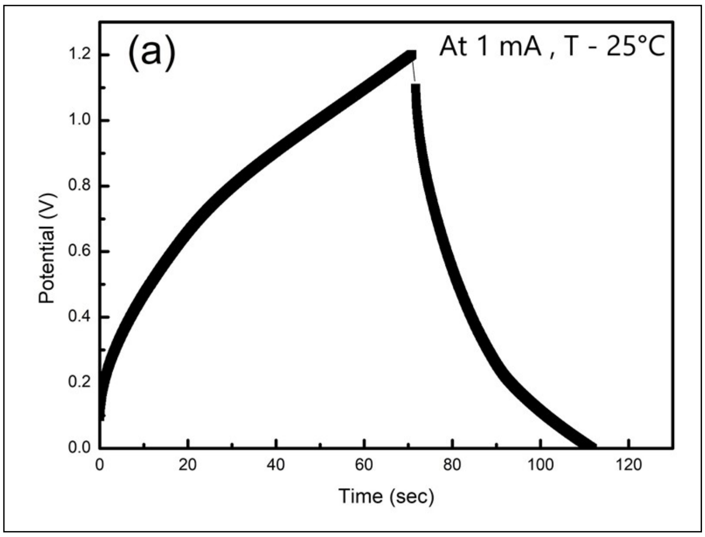

Fig. S-3(a) GCD scans at 1mA for SL-cell without polymer incorporated electrodes. The SL-cells with polymer-added electrodes exhibit substantially low ESR (Fig. 8 (g)).

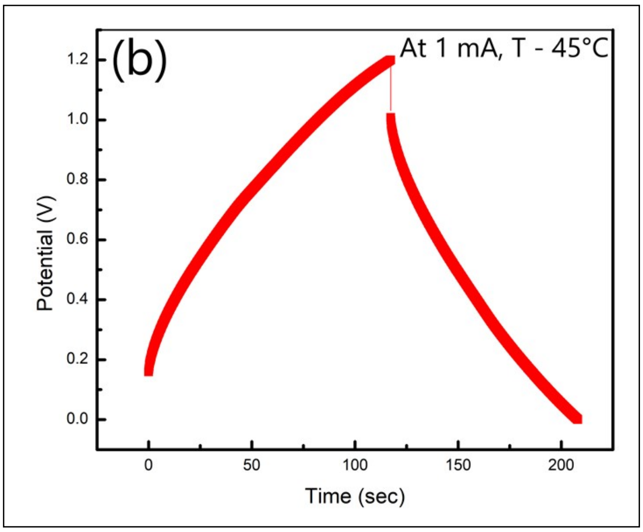

Fig. S-3(b) GCD scans at 1mA for SE-cell without polymer incorporated electrodes. The ESR value is higher than that of the SE-cell with polymer added electrodes. The SL-cells with polymer-added electrodes exhibit substantially low ESR (Fig. 8 (g)).
